# Experimental and Numerical Analysis for the Mechanical Characterization of PETG Polymers Manufactured with FDM Technology under Pure Uniaxial Compression Stress States for Architectural Applications

**DOI:** 10.3390/polym12102202

**Published:** 2020-09-25

**Authors:** Jorge Manuel Mercado-Colmenero, M. Dolores La Rubia, Elena Mata-Garcia, Moises Rodriguez-Santiago, Cristina Martin-Doñate

**Affiliations:** 1 Department of Engineering Graphics Design and Projects, University of Jaen, 23071 Jaen, Spain; jmercado@ujaen.es (J.M.M.-C.); emg00043@red.ujaen.es (E.M.-G.); mrs00025@red.ujaen.es (M.R.-S.); 2 Department of Chemical, Environmental and Materials Engineering, University of Jaen, 23071 Jaen, Spain; mdrubia@ujaen.es

**Keywords:** PETG, FDM, mechanical performance, design, polymeric materials modeling, polymer simulation, Finite Element Method (FEM)

## Abstract

This paper presents the numerical and experimental analysis performed on the polymeric material Polyethylene Terephthalate Glycol (PETG) manufactured with Fused Deposition Modeling Technology (FDM) technology, aiming at obtaining its mechanical characterization under uniaxial compression loads. Firstly, with the objective of evaluating the printing direction that poses a greater mechanical strength, eighteen test specimens were manufactured and analyzed according to the requirements of the ISO-604 standards. After that, a second experimental test analyzed the mechanical behavior of an innovative structural design manufactured in Z and X–Y directions under uniaxial compression loads according to the requirements of the Spanish CTE standard. The experimental results point to a mechanical linear behavior of PETG in X, Y and Z manufacturing directions up to strain levels close to the yield strength point. SEM micrographs show different structural failures linked to the specimen manufacturing directions. Test specimens manufactured along X present a brittle fracture caused by a delamination process. On the contrary, test specimens manufactured along X and Y directions show permanent plastic deformations, great flexibility and less strength under compression loads. Two numerical analyses were performed on the structural part using Young’s compression modulus obtained from the experimental tests and the load specifications required for the Spanish CTE standards. The comparison between numerical and experimental results presents a percentage of relative error of 2.80% (Z-axis), 3.98% (X-axis) and 3.46% (Y-axis), which allows characterizing PETG plastic material manufactured with FDM as an isotropic material in the numerical simulation software without modifying the material modeling equations in the data software. The research presented here is of great help to researchers working with polymers and FDM technology for companies that might need to numerically simulate new designs with the PETG polymer and FDM technology.

## 1. Introduction

Additive technology enables the manufacture of industrial parts, consumer components, and medical products by the addition of material in horizontal layers according to the part geometry. This process makes it possible to obtain a solid model whose surface faithfully reproduces the topology of the designed CAD model. This manufacturing process has multiple advantages over traditional processes such as injection molding or casting [1,2,3], since it manufactures parts impossible to obtain with other conventional technologies. The additive manufacturing process minimizes the waste, thereby reducing the amount of material needed to manufacture the component. The increased use of additive manufacturing technology is essentially due to its adaptability to design changes in product development, to the possibility of product customization according to the customer’s requirements and to the manufacturing of parts with a short delivery time.

There are currently several additive manufacturing processes characterized by using different materials and technologies for material layering. According to the raw materials used for part manufacturing, Additive Manufacturing (AM) processes could be classified as: liquid-based, solid-based and powder-based [4]. The most representative additive processes are material extrusion, vat photo polymerization, sheet lamination, powder bed fusion, binder jetting, material jetting and directed energy deposition [5,6] (see also [7,8,9]). Fused Deposition Modeling technology (FDM) is currently the most widespread manufacturing additive process due to the advantages it offers in terms of manufacturing costs and ease of use [10,11]. Specifically, in the FDM process, components are manufactured from the fusion, extrusion and deposition of plastic material in layers. This process has the advantage of manufacturing complex topologies, although an inappropriate choice of parameters can cause failures in meeting the mechanical and functional requirements of the manufactured part [12].This fact highlights the need to carry out research work in this area and more specifically in the analysis of the plastic material deposition process due to the mechanical requirements and operational specifications of the product [13].

Advanced technologies commonly used in the industrial manufacturing sector [14], are being progressively transferred to the field of architecture and construction. The particularities of the construction field constitute a challenge in the process of adapting technologies from the industrial area. Digital transformation in the construction sector, and in particular the potential of additive manufacturing technologies, can become a key technology for Construction 4.0 [15].

Materials are a very important part of the 3D printing technology as they have to meet the stiffness and strength requirements of the additive manufacturing process. Polymers are optimal for additive manufacturing in construction applications [16,17]. They present as main features low price and low density, it being possible to store them in controlled tanks unlike other materials such as cement.

In the FDM manufacturing process, thermoplastic materials such as PC, ABS and PLA are mainly used due to their low melting temperature [18,19,20]. However, recently, new plastic materials widely used in traditional processes such as injection molding have been introduced into the additive manufacturing process [21,22]. Among these materials, Polyethylene Terephthalate Glycol (PETG) is a polyester thermoplastic, derivative polymer of the material Polyethylene Terephthalate (PET), used for commercial applications such as manufacturing bottles, containers, packaging materials and medical implants [23,24,25]. PETG has excellent formability, durability, chemical resistance and low forming temperature, being an appropriate material for fused deposition modeling, thermoforming and extruding [26,27,28,29,30]. Its main features are resistance against thermal variations, low moisture absorption, resistance to gamma radiation and oxidizing agents, non-slip surface finishing, recyclability and sustainability. All these features make this plastic material suitable for structural and architectural designs located in indoor areas. In terms of manufacturing, PETG is a versatile material with low printing requirements. Its extrusion temperature reaches 235 °C. The recommended temperature of the 3D printer hot bed is 70 °C. Several authors have analyzed the mechanical properties of the PETG material, studying the influence of the printing parameters on the FDM manufacturing process. Guessasma et al. [31] presented an analysis of the manufacturability and performance of FDM 3D printed PETG material in tensile tests. Khan et al. [32] analyzed the influence of the filling pattern on the tensile behavior of the PETG material manufactured with FDM, finding that the concentric manufacturing pattern produced a better elongation compared to the rectilinear pattern, which presented more strength. Dev Singh et al. [33] studied the tensile behavior for FDM printed PETG material in comparison with glass fiber loaded PETG material, obtaining better results for the loaded material. Hanon et al. [34] carried out research with PETG material printed in FDM, analyzing the performance of the PETG material in comparison with previous results in elongation for break tests.

Large-scale construction processes are particularly vulnerable to the lack of accuracy, and for this reason plastic materials have been appropriately considered for additive construction [16]. Recycled plastic materials are widely used in the AM process. Waste plastic filaments, misprints and undesired outputs can be reclaimed and reused. This could be an enabler and a driving force for improved construction sustainability [17]. Although in recent years the application of plastic materials in additive manufacturing has had a conceptual character, at the present moment, FDM technology evolves from rapid prototyping towards a rapid manufacturing method, changing the main purpose in producing finished components [35]. There are several approaches to the use of plastic materials in additive architectural construction [36,37]. Unfortunately, despite the advantages of the use of plastic materials in additive manufacturing for architectural purposes, they have been used in very few applications [16].

The numerical validation of the mechanical behavior of polymeric components manufactured with FDM implies knowing in advance the anisotropic behavior of the printed polymer according to the manufacturing specifications and to the final design requirements. This research information is highly important for companies and researchers but currently it is not available because suppliers provide data sheets that only include technical specifications of standard filaments. The FDM manufacturing process transforms considerably the elastic and mechanical properties of the polymer due to the heat treatments and the external stresses that occurs during the process. To obtain the mechanical behavior of FDM manufactured polymers for numerical simulations, it is required to perform several experimental tests using a set of specimens manufactured according to the requirements of the standard. The results of the experimental tests for characterizing polymers are expensive in terms of time and money. Additionally, to complete the numerical simulation once the experimental information has been obtained, it is necessary to find the best way to treat and configure the experimental information in the FEM software in order to obtain simulation results that perfectly match the material experimental behavior. For the case of the PETG polymer subject to uniaxial compression tests, there are no specific scientific or industrial data that allow characterizing the material in the FDM process for specific manufacturing requirements from technical product specifications.

The research level most of the studies that have been carried out so far with other polymers analyzes the relation between FDM process parameters and the mechanical behavior only for test specimens. However, for industrial and construction uses, it is necessary to know if the mechanical behavior obtained from experimental tests using 3D printed specimens could be used for predicting the mechanical behavior of the FDM end-parts. According to Popescu et al. [38], there are very few research papers that study and demonstrate a mechanical characterization of polymers manufactured with FDM with test specimens and parts, and at the same time configure a real and accurate behavior of the anisotropic manufactured polymer for numerical simulations. This area of research is considered as a new line of research in polymers.

Numerical simulation programs do not have updated mechanical characterization models of the materials used with FDM technology [39]; they only include information regarding the mechanical parameters of the plastic material filaments. This does not properly represent the mechanical behavior of the final printed plastic part. The usual ways that Finite Element Analysis (FEA) is employed may not be applicable in FDM processes due to the inherent anisotropy and uncertain qualities of 3D-printed parts. FEA can no longer accurately predict the behavior of 3D-FDM printed parts in the same way it estimates the behavior of parts produced by traditional methods. What adds to the complexity are the different additive manufacturing (3D printing) methods and the lack of test results of using these printing methods, and therefore the existence of very few data regarding strength [39].

Additionally, the use of FDM additive manufacturing in construction and specifically using new materials such as polymers is presented as a key pillar in the field of construction 4.0 in the coming years [16,17].

However, despite their impact and imminent demand in the construction field for more complex designs manufactured with polymers in FDM additive environments, there is no methodology currently capable of characterizing the mechanical behavior of the PETG polymer subjected to uniaxial compression loads and FDM additive technology for numerical 4.0 environments.

To solve these problems, this paper presents an experimental and numerical analysis whose objective was to obtain the elastic and mechanical properties of PETG plastic material manufactured with FDM technology in three main manufacturing directions and under states of pure uniaxial compression stress. Additionally, the research presented here validates the mechanical characterization of the PETG material on a free-form structural element manufactured with FDM technology in two main manufacturing directions and according to the design requirements of the real environment in which it will be located. This presented research is of great help to researchers who work with polymers and FDM technology or companies that might need to numerically simulate new designs with the PETG polymer and FDM technology. The methodology presented in this paper is currently capable of characterizing the mechanical behavior of the PETG polymer subjected to uniaxial compression loads and FDM additive technology for numerical 4.0 environments as an isotropic material in the numerical simulation software without modifying the material modeling equations in the data software. These results allow us to reach conclusions which very few research papers can emulate, since most of the research presented shows only general results in the test samples [39]. The results of our research point to the use of PETG material as promising in FDM manufacturing processes and especially for architectural applications.

## 2. Materials and Methods

### 2.1. Geometrical Design and Analysis for the Constructive Element Manufactured through the FDM Process

**Figure 1:**
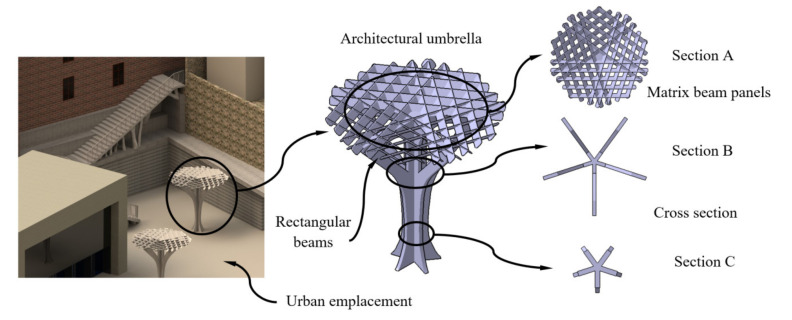
Urban location and topology of the architectural umbrella.

In this section, the geometrical, functional and manufacturing features associated with the construction element under study are described. The structural element is defined as an innovative architectural umbrella (see Figure 1) manufactured using FDM technology and plastic materials. Technical details regarding the selection of the plastic material for the additive manufacturing process, boundary conditions and load scenario associated with the mechanical and structural state of the structural element are also specified.

The free-form structural topology (see Figure 1) presents cross-shaped geometry in the base reinforced with four bracket supports in the highest area (see Figure 1). The top of the architectural umbrella comprises a panel array with a pentagonal shape (see Figure 1) whose objective is to reduce the incidence of the light rays on the urban pavement. The topology of the structural element has been designed with symmetry, providing both structural efficiency and aesthetic adaptation to the urban environment.

**Figure 2:**
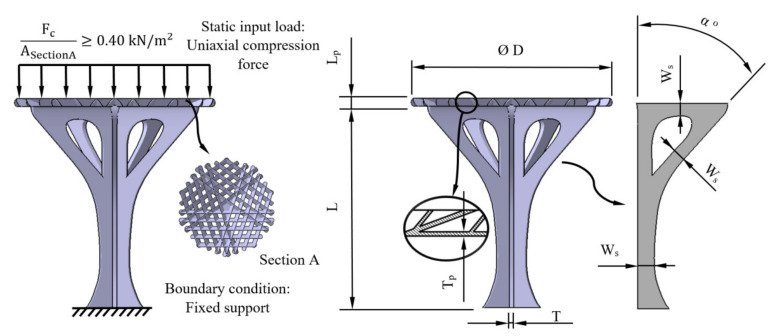
Boundary conditions, load definition and main geometrical parameters of the plastic structural element.

According to the Spanish CTE technical building code [40], the structural element under study is subject to a uniaxial pure compression stress state (see Figure 2). This stress state is defined by a snow load applied on the upper surface of the structural element (see Figure 2). The load magnitude is determined by the environment requirements of where the structural element will be framed.

The main objective of this manuscript is the experimental and numerical analysis of the mechanical behavior of the structural element under study, as well as the characterization of the elastic and mechanical properties of the PETG plastic material, assuming for both cases a uniaxial pure compression stress state (see Equation (1)).

(1) $$\sigma_{c}=\int_{L_{o}}^{L}\frac{F_{c}}{A_{\mathrm{cross}}\left(z\right)}\cdot \mathrm{dz}$$

**Figure 3:**
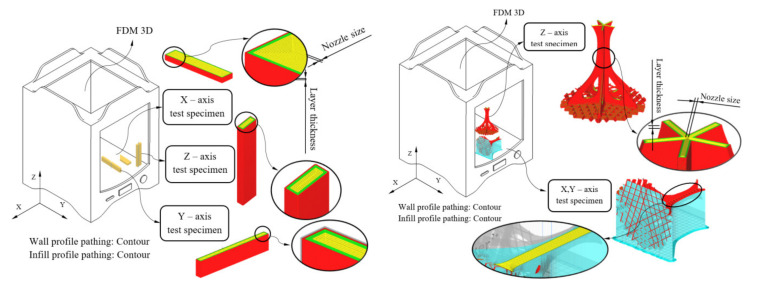
FDM process configuration for test specimen and the structural element manufacturing.

The tensile compression stress of the structural element σ_c_ (MPa) is defined by the cross section of the element geometry A_cross_ (mm^2^), the uniaxial compression force F_c_ (N) and the part length L along the longitudinal axis. As shown in Figure 3, the magnitude of the pressure on the structural element, according to the Spanish CTE technical building code [40], must be greater than 0.40 kN/m^2^, and it is applied to the top section of the architectural umbrella, which is defined as section A. Furthermore, the boundary condition is a fixed support applied to the bottom section of the architectural umbrella, the region in contact with the ground.

The main dimensions of the structural element have been calculated according to its functional, manufacturing and urban environmental requirements. Since the manufacturing of the structural element was carried out using FDM technology, the geometry and the magnitude of the design parameters were adapted to optimize the manufacturing process and minimize the volume of auxiliary supports. The use of a recyclable polymer such as PETG in the manufacturing process provides sustainability features to the proposed design. Table 1 and Figure 2 show the main geometrical parameters that establish its topology.

Table 2 shows the dimensions of the structural element on a scale 1:1. These dimensions would present difficulties in carrying out an experimental test of the part using a standard compression test device. For this reason, a dimensional reduction scale was applied, of magnitude 1:25, on the structural element dimensions (see Table 2).

### 2.2. Additive Manufacturing 3D Process of the Constructive Element under Study

The structural element under study (see Figure 1) was manufactured using FDM technology and the thermoplastic material PETG. Table 2 shows the magnitude of its physical and technological variables. These parameters allow high adhesion between layers, decreasing defects originated for an excess of viscosity and causing stress concentrations between layers. These magnitudes were defined on the basis of the experience and recommendations of the material manufacturer [41].

The manufacture of the structural element was carried out using an FDM additive manufacturing process and a 3D printer device, (Ultimaker 2+, Ultimaker, Utrecht Netherlands) [42]. The dimensions and manufacturing volume for this 3D printer are: 223 (X-axis), 223 (Y-axis) and 205 mm (Z-axis). From a structural point of view, this manufacturing process must guarantee the safety of the parts manufactured or, at least, achieve a mechanical strength similar to that obtained with conventional manufacturing processes (for instance, injection molding). The mechanical and elastic properties of the resulting geometry after the 3D printing process mainly depend on the previous features of the plastic material and on the technological parameters applied during the manufacturing process. To take full advantage of the FDM process, it is convenient to align the manufacturing direction that presents the greatest mechanical strength with the direction of the forces and stresses to which the part will be subject. Therefore, to evaluate the printing direction with the greatest mechanical strength for the structural element manufactured using PETG material, eighteen test specimens with prismatic geometry and rectangular cross section have been built (six on X-axis, six on Y-axis and six on Z-axis, see Figure 3) according to the requirements of the ISO-604 standard (2003) [43]. The specimens have been manufactured from top to bottom following the positive axis direction of the FDM printer. The possibility of analyzing the influence of the printing direction on the mechanical and elastic properties of PETG concretely under uniaxial compressive stress states presents an important asset for industrial and construction applications.

**Figure 4:**
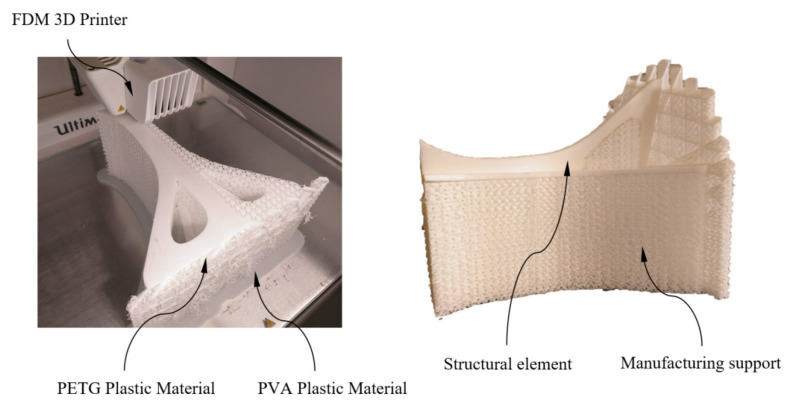
Manufacturing of the structural element under study.

Two prototypes of the mechanical element under study were manufactured at a 1:25 dimensional scale (see Figure 3 and Figure 4). For both prototypes, the main 3D manufacturing direction was aligned with the longitudinal direction of the structural element (coinciding with the Z-axis direction) and with the orthogonal direction to the element longitudinal axis (coinciding with the X- or Y-axis direction). It should be noted that, since the geometry of the structural element presents symmetry of revolution along its longitudinal axis, its mechanical and elastic behavior is analogous for the main X and Y directions of 3D printing. In this way, the use of two prototypes is justified to optimize technological resources and verify that the mechanical and elastic properties obtained experimentally for the specimens printed in the X- and Y-axis direction are valid for characterizing the mechanical and elastic behavior of the structural element in the orthogonal direction to its longitudinal axis. Polyvinyl alcohol (PVA) plastic material has been required for manufacturing supports in an orthogonal position of the component (coinciding with the X- or Y-axis direction). PVA has the property of being soluble in water, thus the supports are easily removed (see Figure 3 and Figure 4) after submerging for 24 h in water at room temperature.

As shown in Figure 3, the fill pattern used for manufacturing the specimens and the structural element was contour profiles. This pattern was applied to both layers corresponding to the outer wall and layers corresponding to the internal filling pattern. Table 3 shows the technological parameters used during the manufacturing process, for prismatic specimens and prototypes of the structural element under study. Product mechanical requirements for the manufactured part were adapted to the specifications of the FDM process.

### 2.3. Experimental Tests of the Specimens

Two experimental tests were performed, aimed at analyzing the structural and mechanical behavior of the geometry under study and obtaining the mechanical and elastic properties of the PETG manufactured thermoplastic material using an FDM process. Firstly, an experimental test according to the requirements established in the ISO-604 standard (2003) [44] allowed us to obtain the elastic and mechanical properties of the PETG plastic material subjected to a uniaxial pure compression stress state. Next, a second experimental test analyzed the mechanical behavior of the structural element (see Figure 1) under a pure uniaxial compression stress state according to the Spanish CTE technical building code [40]. The experimental results of the test specimens allow establishing the mechanical and elastic features of the PETG plastic material as compression yield stress, displacements and loads until reaching the compression yield stress, compressive stiffness and ultimate yield stress. Secondly, the elastic and mechanical parameters obtained from the experimental tests were used to model the mechanical FEM simulations for analyzing the geometry under study. In this way, it was possible to compare the results obtained from the numerical simulations and those obtained from the experimental tests, validating the methodology presented in this paper. The proposed methodology defines and models the elastic and mechanical behavior of the thermoplastic material PETG manufactured with FDM in order to use it in numerical simulations.

The elastic and mechanical characterization of the PETG was performed according to the requirements and methodology of the ISO-604 standard for plastic materials under uniaxial compression stress states. The standard ISO-604 establishes that the experimental tests for material characterization must use specimens with prismatic geometry. To evaluate the printing direction which presents greater mechanical strength and performance, specimens were manufactured in X, Y and Z directions, obtaining technical values in these three building directions. Table 4 shows the magnitude of the variables that parameterize the geometry of the prismatic specimens. These dimensions were used for calculating the uniaxial compression elastic module and the compression yield stress.

**Figure 5:**
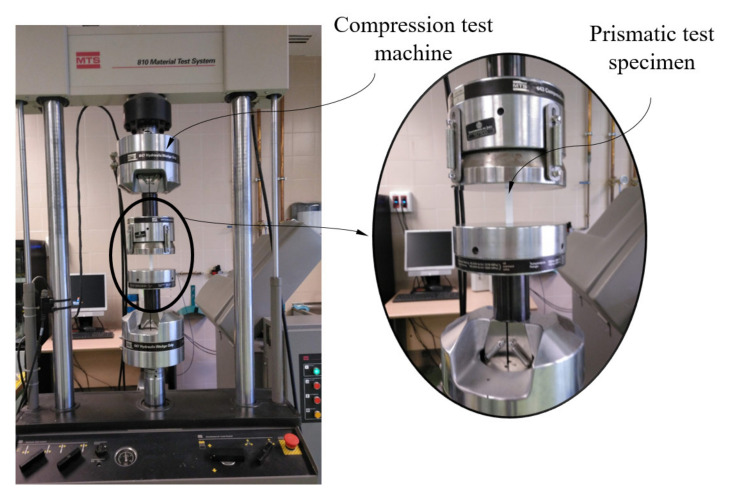
Test of uniaxial compression for specimens, testing machine MTS-810.

According to the ISO-604 standard, at least five specimens must be tested in each manufacturing direction. As Figure 3 shows, the main 3D printing directions were X, Y and Z. Therefore, 18 specimens (six for each direction of analysis) were built with the same manufacturing parameters as those of the structural element under study. To perform the experimental test, once all the specimens were manufactured, the flat end surfaces of the prismatic specimens were placed on the flat jaws of the test machine. Figure 5 shows the location of the specimens in the test machine during the experimental tests. This positioning was established to maintain a parallelism between the jaws and the flat surfaces of the specimens. Before starting the experimental compression test, the testing machine performed an adjustment of its jaws at a low compression speed to center the specimens and avoid eccentricities. This fact added flexural–compression stresses to the mechanical behavior of the specimens during the test, which were intended to be avoided. On the other hand, according to ISO-604, the compression speed from the beginning until the moment of the specimen breakage is constant and equal to 1 mm/min (see Equation (2)). That means the experimental compression tests of all prismatic specimens began with the jaws’ adjustments movement of the test machine, continued with the specimen deformation at a speed of 1 mm/min and finalized with the specimen breakages.

(2) $$v_{c}=0.02\cdot L_{s}$$

where v_c_ (mm/s) characterizes the compression speed of the test and L_s_ (mm) is the length of the plastic specimens tested. The test machine used to carry out the experimental tests is MTS-810 (see Figure 5), which meets the requirements established in the ISO-5893 standard. The MTS-810 test machine is servo hydraulic, having two hardened steel compression jaws parallel to each other on a plane perpendicular to the direction of application of the compression force. Similarly, this test machine includes force transducer measuring devices to visualize and store the forces and displacements for the geometries analyzed during the experimental tests. Table 5 shows the technical specifications of the test machine used for the experimental tests.

### 2.4. Experimental Tests of the Structural Element under Study

**Figure 6:**
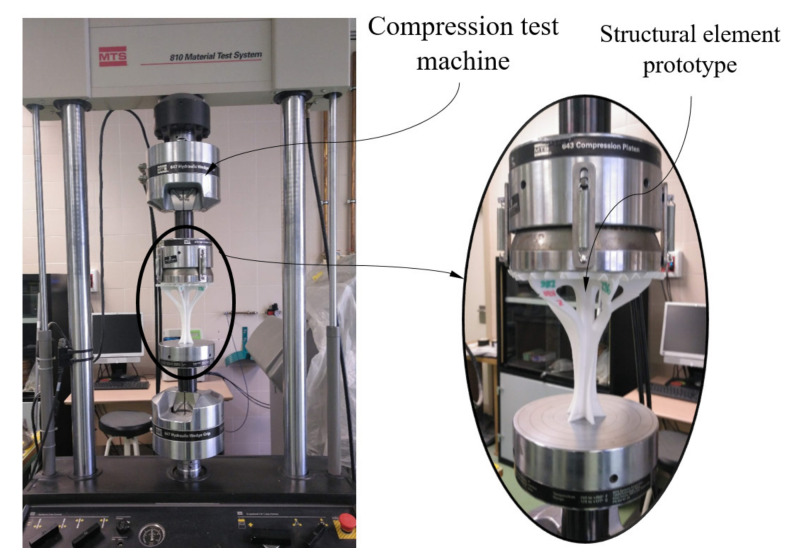
Uniaxial compression experimental test for the part under study.

After performing the tests of the prismatic specimens to characterize the PETG material, series of experimental tests were carried out on the structural element under study. These experimental tests allowed analyzing the mechanical behavior of the structural element, taking into account the loads and boundary conditions according to the standards. The structural element was manufactured using FDM in X and Z directions (see Figure 3), maintaining, in both cases, the manufacturing parameters shown in Table 3. In this way, it was possible to evaluate and compare the influence of the building direction on the mechanical behavior of the geometry under study. The uniaxial compression tests performed on the structural element prototypes were carried out on an MTS-810 test machine (MTS Systems Corporation, Eden Prairie, MN, USA) (see Figure 6) using a compression speed constant and equal to 1 mm/min. This compression speed was analogous to the compression speed used in the tests of the prismatic specimens, since the results of both tests must be comparable.

### 2.5. Numerical Method

**Figure 7:**
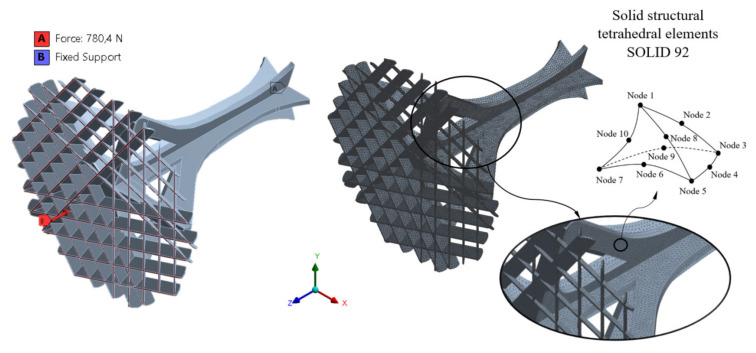
Boundary requirements and loads and mesh generated for the numerical simulations of the structural element.

The commercial FEM numerical calculation software used to perform the numerical simulations of the structural element under study was Ansys Mechanical (ANSYS, Inc, Canonsburg, PA, USA) [45]. Figure 2 and Figure 7 show the boundary conditions and load scenario defined for the numerical calculation of the geometry mechanical behavior. On the one hand, the load scenario was established as a uniaxial compression force, with a direction coincident with the longitudinal axis of the structural element (Z-axis, see Figure 7) applied to its upper surface and with a magnitude equal to 780.4 N according to the functional design requirements. The boundary conditions were defined as a fixed support or embedment in the base surface of the structural element and coincident with the surface of the ground where it is located (see Figure 7).

The mechanical model used for defining the numerical analysis was elastic and linear. Three numerical simulations were performed for the geometry of the structural element to analyze its mechanical behavior along the manufacturing directions X, Y and Z. The main objective of these simulations was to validate the presented methodology by defining the mechanical and elastic properties of the PETG material in the numerical software and verify the numerical results with the experimental ones. The PETG plastic material was defined in the numerical analysis as isotropic, elastic and linear, with a Poisson coefficient of magnitude 0.38 (value defined by the material manufacturer) [41], compression Young’s modulus constant and equal to 1329.5, 1117.9 and 1124.0 MPa, respectively, for the primary X, Y and Z manufacturing directions.

Solid structural tetrahedral units SOLID 92 were used to discretize the structural geometry. Each tetrahedral unit was presented by a quadratic displacement made up of 10 points where four nodes were located in the vertices of the tetrahedron and six points in the midpoints. Each point had three degrees of freedom with translation in the nodal X, Y and Z directions. To obtain the size of the units, a sizing procedure was defined, resulting in a section size of 1.5 mm. Table 6 presents the statistics for the numerical simulations performed and Figure 7 shows the mesh. Based on the model of the PETG presented for the numerical simulation, the large displacement option in the initial solver definition was used to ensure the convergence of the final solution.

## 3. Results and Discussion

### 3.1. Experimental Results and Discussion of the Prismatic Specimen Compression Tests

**Figure 8:**
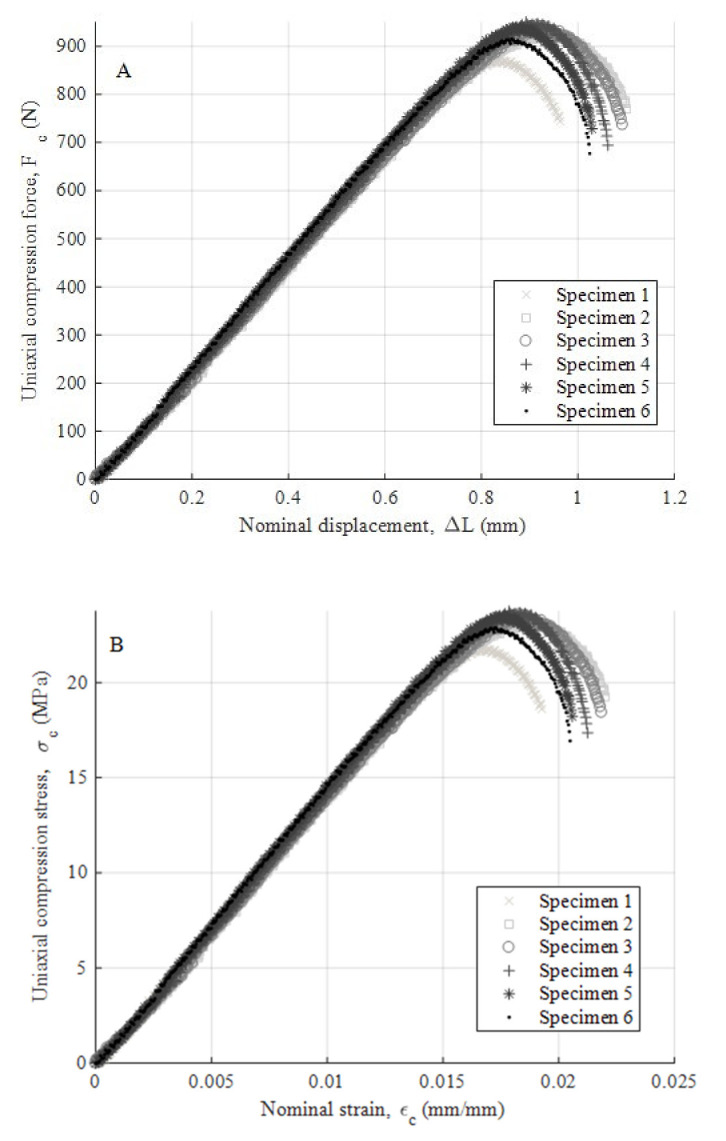
Curves for Z-axis specimens related to compression force versus nominal displacements (**A**) and compression stress versus nominal strain (**B**).

**Figure 9:**
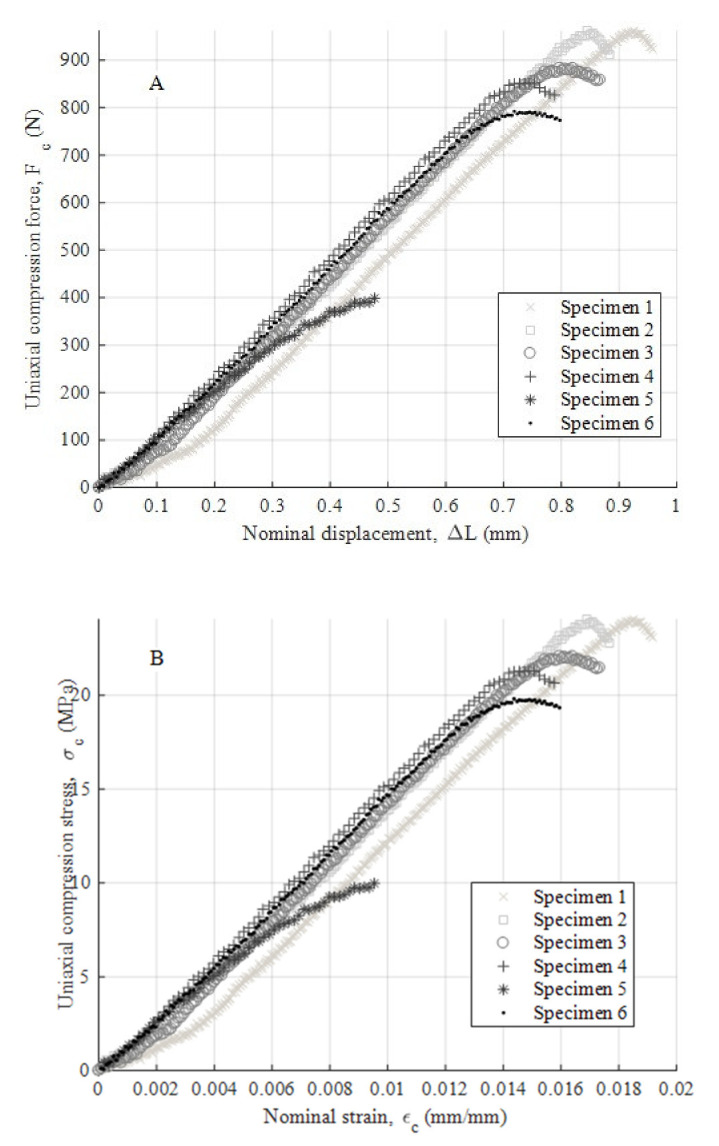
Curves for X-axis specimens related to compression force versus nominal displacements (**A**) and compression stress versus nominal strain (**B**).

**Figure 10:**
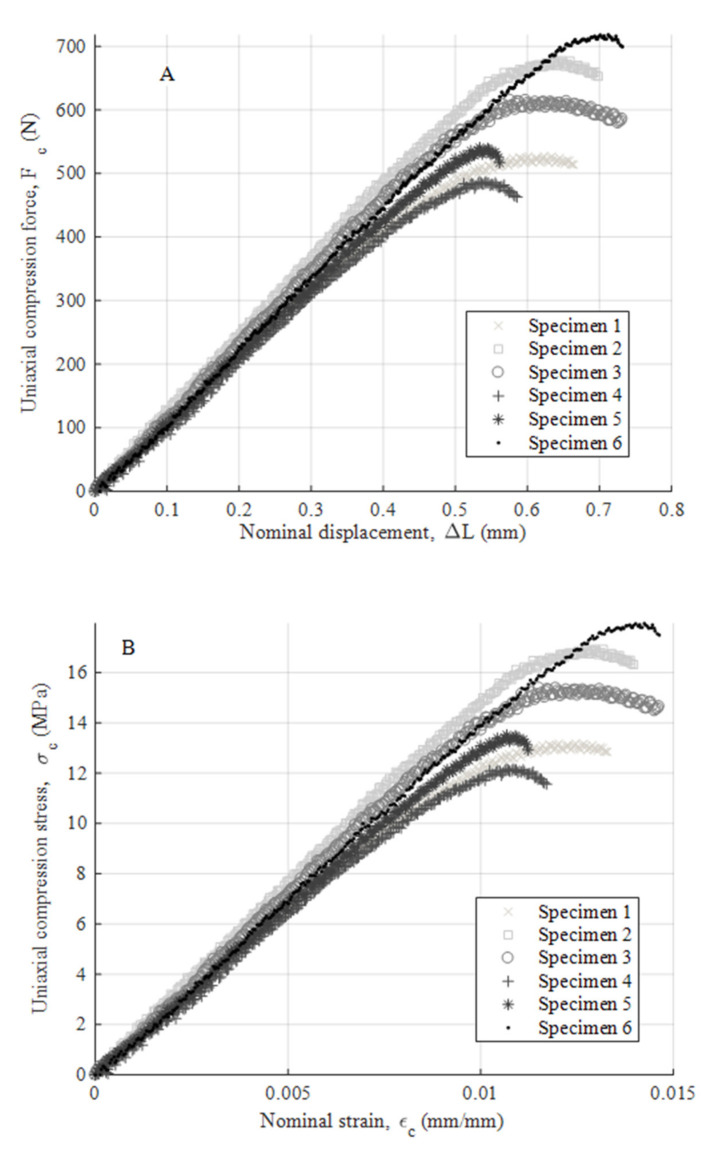
Curves for Y-axis specimens related to compression force versus nominal displacements (**A**) and compression stress versus nominal strain (**B**).

After performing the experimental mechanical test, Figure 8a,b, Figure 9a,b and Figure 10a,b show the experimental results obtained from the uniaxial compression forces versus the displacements field along the load direction for the test specimens manufactured in the three main directions, the X-, Y- and Z-axis, respectively. These experimental results cover the whole test, that is from the beginning of the test to the moment of breakage or mechanical failure of each test specimen. From these experimental results, the field of uniaxial compression stresses and nominal strains can be determined, according to the methodology described in the ISO-604 standard (see Equations (3) and (4)).

(3) $$\sigma_{c}=\frac{F_{c}}{A_{\mathrm{cross}}}$$

(4) $$\varepsilon_{c}=\frac{\Delta L}{L_{S}}$$

where σ_c_ (MPa) represents the field of uniaxial compression stresses on the test specimens, F_c_ (N) represents the field of uniaxial compression forces on the test specimens, A_cross_ (mm^2^) represents the cross-sectional area of the test specimens (this parameter is considered constant during the compression test), ε_c_ (mm/mm) represents the nominal compression strain on the test specimens along the load application direction and ΔL (mm) represents the field of displacement on the test specimens along the load application direction.

As shown in the stress–nominal strain curves (σ_c_–ε_c_) (see Figure 8, Figure 9 and Figure 10), the mechanical behavior of the PETG used for the manufacture of test specimens in the directions X, Y and Z is completely linear from the beginning of the curve until it reaches the yield strength at a compression value σ_y_ (MPa) (see Table 7, Table 8 and Table 9). For this magnitude of stress, the process of plasticizing the test specimens begins until they reach the moment of breakage or fracture stress σ_f_ (MPa). The structural failure is characteristic and varies depending on the main direction in which the specimens have been manufactured. On the one hand, for manufacturing direction Z, the fracture process is caused by a delamination of adjacent welded layers beside a brittle fracture of polymer filaments. This is caused by the field of flexural stresses to which the specimens are subjected during the deformation and plasticization hardening phase. On the other hand, for manufacturing directions X and Y, the specimens undergo a permanent plastic deformation that produces the collapse and structural failure of the material. The specimens do not undergo a crack growth process due to the flexibility and lower structural stiffness of the material. This behavior causes an elastic and plastic deformation up to the moment of structural failure without generating an apparent fracture in the outer layers of the plastic material. Furthermore, for these cases, the fracture point is located in the central cross sections of each specimen and the stress corresponding to the fracture point is considered as the yield stress fracture. According to the ISO-604 standard, the compression Young’s modulus has been defined from two values of the nominal stress–strain curve (σ_c_–ε_c_). As Equation (5) shows, these values are established for a nominal strain magnitude equal to 0.0025 and 0.0005, respectively.

(5) $$E_{c}=\frac{\sigma_{c}\left(\varepsilon_{c}=0.0025\right)-\sigma_{c}\left(\varepsilon_{c}=0.0005\right)}{0.0025-0.0005}$$

where E_c_ (MPa) represents the Young’s modulus of compression of the PETG plastic material. Table 7, Table 8 and Table 9 show the magnitude of the Young’s modulus obtained for each direction of analysis (X, Y and Z, respectively). These values were determined as the arithmetic mean of the elastic modulus obtained from the test specimens. In addition, Table 7, Table 8 and Table 9 show the magnitude of the yield stress (stress that determines the yield limit) and fracture stress (stress that determines the moment of collapse or structural failure) for each direction (X, Y and Z, respectively).

The test specimens manufactured in the Z direction show a brittle fracture with similar elastic and fracture limits. These values indicate that the point at which the plastic material begins to plasticize is close to the breaking moment with a very short plasticizing process. The fracturing process is mainly determined by the manufacturing process causing delamination between adjacent layers of welded material. On the other hand, test specimens manufactured in the X and Y directions present a structural failure when the uniaxial compression stress is greater than the elastic limit. There is a ductile process of plastic hardening of the material and permanent deformation of the same. However, in both cases, the structural collapse of the material occurs in the central sections of the specimens, which is caused by a field of bending stresses during the structural failure process.

### 3.2. Experimental Results and Discussion of the Structural Element under Study Compression Tests

**Figure 11:**
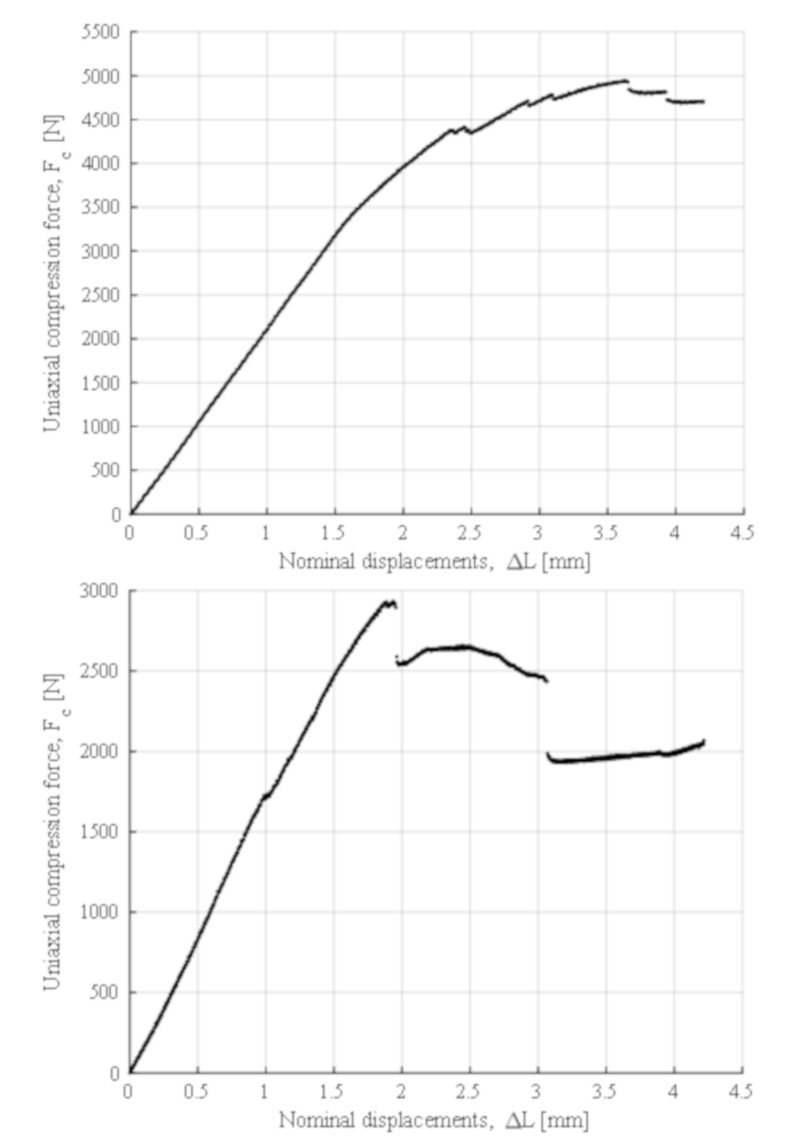
Uniaxial compression load versus nominal displacements for the Z (**top**) and X/Y (**bottom**) axis elements tested.

Furthermore, since the aim of the present manuscript is to evaluate the mechanical behavior of the structural element under study, Figure 11 shows the plots of uniaxial compression force against the nominal field of displacement (F_c_–ΔL_c_) obtained from the experimental tests of the structural element manufactured in directions Z and X/Y, respectively. The plots represent the elastic and mechanical behavior of the structural element from the experimental tests results until the structural failure point of the geometry. Table 10 and Table 11 show the maximum uniaxial compression force and its corresponding nominal displacement for the fracture point. Considering the curves of uniaxial compression forces versus nominal displacements (F_c_–ΔL_c_) (see Figure 11), it can be seen that the structural failure of the element under study is different both in the magnitude of the maximum values and in the mechanical fracture typology.

**Figure 12:**
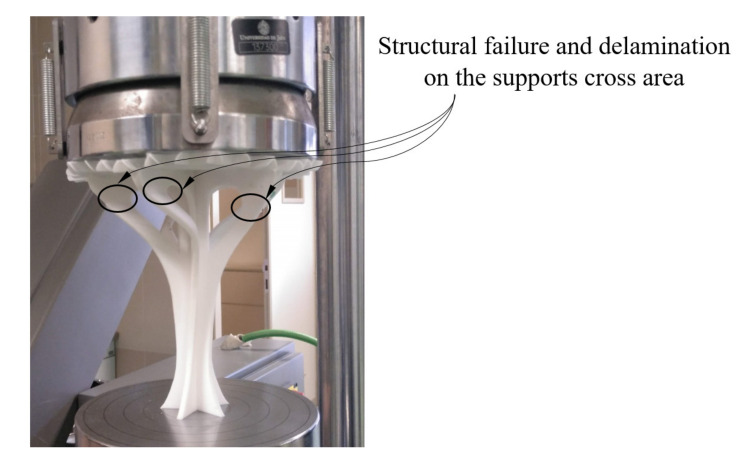
Fracture of the Z-axis printed element under study.

**Figure 13:**
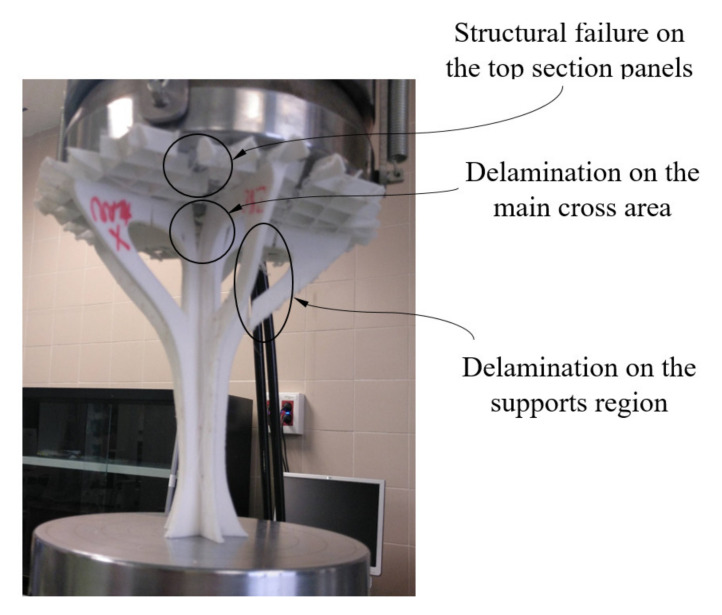
Fracture of the X–Y-axis printed element under study.

The fracture process of the structural element manufactured in the Z direction (see Figure 12) is given by the delamination and breakage of the plastic filaments of adjacent layers located in the supports (see Figure 4). This type of fracture is not completely brittle; the plastic material undergoes a hardening process by plasticization in the areas where fractures propagate (see Figure 12). In this way, it can be verified that the elastic and mechanical behavior of the prototype printed in the main Z direction is linear, until reaching both a uniaxial compression force of 3300 N and a nominal displacement of 1 mm. From this point, the material plasticization begins in the support area until it breaks under a uniaxial compression force of 4700 N and with a nominal displacement of 4 mm. The fracture process for the prototype printed in the X direction is sequential and linked to the different areas of the structural element (see Figure 13). Firstly, the elastic and mechanical behavior of the structural element is linear until reaching a uniaxial compression force of 2900 N and a nominal displacement of 1.9 mm. At this magnitude, the top panels collapse, causing the structural failure of the geometry under study. From these values, and despite the loss of mechanical strength, the fracture is transmitted through the central area of the structure. This fracture produces delamination between adjacent layers, causing separation in the five supports of the structural element (see Figure 13) and an ultimate structural failure of the geometry.

The structural element under study must resist mechanically a uniaxial compression pressure on its upper surface of at least 0.4 kN/m^2^ according to the of the Spanish CTE technical building code. From the required pressure and its application area, it is possible to obtain the total uniaxial compression force in order to validate the structural element, this value being 780.4 N. From the experimental results (see Table 10, Table 11 and Figure 11), it is possible to assure that the structural safety of the element will not be engaged in any case for the boundary requirements and mechanical forces to which it will be subjected. This is due to the fact that the maximum uniaxial compressive forces that the prototype supports are 4942.0 and 2930.0 N in Z and X–Y manufacturing directions, respectively, far above the value required by the of the Spanish CTE technical building code. This fact validates the use of the PETG material manufactured through the FDM process according to the operating requirements presented.

### 3.3. Comparison between Experimental and Numerical Results of the Structural Element under Study

**Figure 14:**
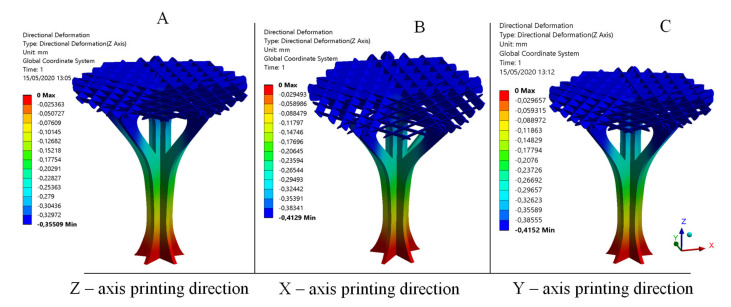
Field of displacements: (**A**) Z-axis printing direction; (**B**) X-axis printing direction; and (**C**) Y-axis printing direction.

**Figure 15:**
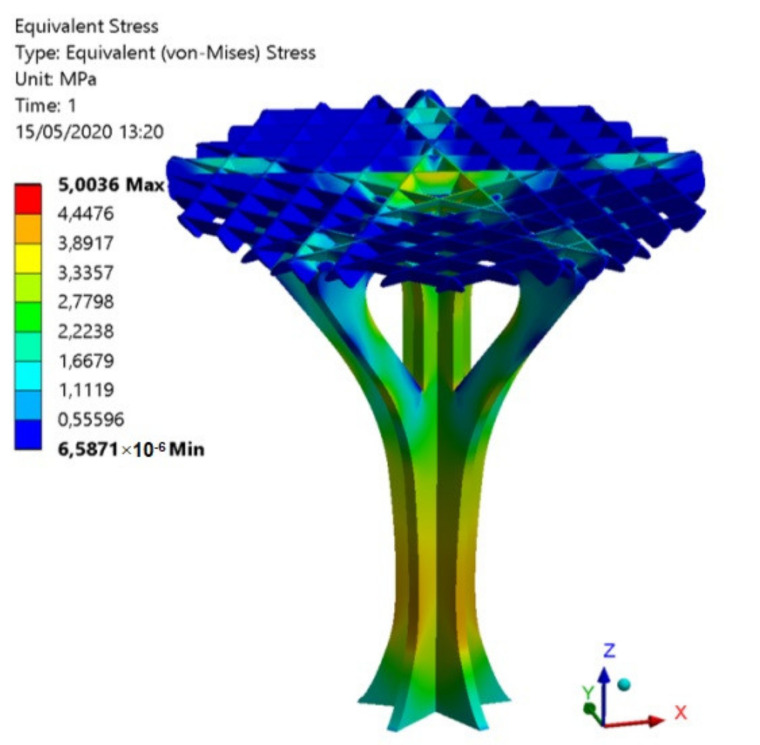
Field of von Mises stress obtained in the mechanical simulations.

The comparison between the experimental and numerical results is one of the objectives proposed in this manuscript as well as the validation of the numerical modeling used to define the elastic and mechanical properties of the PETG for FDM additive manufacturing. As shown in the numerical results (see Figure 14 and Figure 15), the structural element manufactured in the Z direction presents a nominal displacement of 0.354 mm and a stress state of 5.00 MPa for a uniaxial compression force of magnitude 780.4 N using the elastic and mechanical properties for PETG manufactured in Z direction. Secondly, the structural element manufactured in the X direction presents a nominal displacement of 0.412 mm and a stress state of 5.00 MPa for a uniaxial compression force of magnitude 780.4 N using the elastic and mechanical properties for PETG manufactured in the X direction (see Figure 14 and Figure 15). Finally, the structural element manufactured in the X direction presents a nominal displacement of 0.414 mm and a stress state of 5.00 MPa for a uniaxial compression force of magnitude 780.4 N using the elastic and mechanical properties for PETG manufactured in the Y direction. Thus, it can be said that the structural behavior of the structural element prototypes under the boundary stress conditions to which they will be subjected is far from collapse or fracture.

Figure 14 shows the nominal displacement field from the numerical simulations using the elastic and mechanical properties for PETG manufactured in the Z, X and Y directions. The numerical analysis using the parameters from characterization of the PETG material manufactured in Z presents a maximum nominal displacement in the direction of load application of 0.354 mm. In the second numerical simulation performed using the PETG characterization for elements manufactured along the X manufacturing direction, the maximum displacement (in the direction where the load is applied) was 0.412 mm. The third numerical simulation, where the PETG plastic material was characterized along the Y manufacturing direction, presented a maximum displacement (in the direction where the load is applied) of 0.414 mm. The relation between numerical and experimental results presents a percentage of relative error of 2.80% (Z-axis), 3.98% (X-axis) and 3.46% (Y-axis), respectively (see Table 12). These results validate the numerical model presented as a useful evaluation tool for PETG plastic material, as well as for the structural behavior of the printed structural element.

Based on the elastic properties resulting from the tests based on the ISO-604 standard, it can be stated that the PETG material manufactured using FDM processes can be characterized as an isotropic material for the structural simulation of parts under uniaxial compression loads. The numerical analysis of isotropic polymers does not present complexities in the FEM software since it does not require changes in the characterization of the material properties in the data base. Finally, it does not require information on the positioning of the material in the simulation model. For simulations of the material outside the elastic behavior zone, the material is anisotropic, and the incorporation of these characteristics into the software is necessary.

Finally, according to the ISO-604 standard, and in order to validate the experimental test performed with plastic material test specimens, the maximal value of nominal deformation to characterize the elastic properties of PETG material under compression loads should accomplish Equation (6).

(6) $$\varepsilon_{c}^{*}\leq 0.4\cdot \frac{T_{s}^{2}}{L_{s}^{2}}\to 0.0025\leq 0.4\cdot \frac{4^{2}}{50^{2}}=0.0026$$

The geometrical features of the specimens used for characterizing the PETG material are presented in Equation (6), where T_s_ (mm) makes reference to the specimen thickness, $\varepsilon_{c}^{*}$ to the nominal maximum deformation for obtaining the elastic properties of PETG and L_s_ (mm) to the specimen length. The ISO-604 standard requirements were fulfilled since the maximum nominal deformation that characterizes the compression Young’s modulus was 0.0025, less than the 0.0026 value of deformation presented in the inequality of the Equation (6).

### 3.4. Fractography

To analyze the mechanical fractography of the test specimens, a high-resolution scanning microscope (FESEM) was used. The microscope model was Carl Zeiss (Merlin- Carl Zeiss, Oberkochen, Germany) with analytical capacity EDX and WDX Oxford, a hot-tip laser cannon for the emission of the electron field, secondary electron detectors of high resolution located in the sampling chambers (SE Everhart-Tornley and SE in lens), high-resolution back-triggered electron detectors located in the sampling chambers (4-quadrant solid state AsB integrated into the lens of the GEMINI II column and EsB in lens) and cathodoluminescence detectors (CL). This microscopy device offers maximum imaging resolutions of 0.8 nm at 15 kV, 1.4 nm at 1 kV and 2.4 nm at 0.2 kV. Furthermore, the magnitude of the potential acceleration range is between 0.02 V and 30 kV, and the current of the electron beam is between 10 pA and 300 nA.

The chemical microanalysis mechanism consists of an EDX X-ray detector (Oxford Inca Energy 350X-MAX 50, Oxford Instruments, Abingdon, Oxfordshire, UK) with a linear resolution of 127 eV in Mn Kα from 1 to 100,000 cps. This is complemented, in turn, with a WDX spectrometer, Oxford Inca Wave 500 (Oxford Instruments, Abingdon, Oxfordshire, UK), with a Rowland circle of 210 mm and 2θ range from 33° to 135°, including four analyzer crystals with six interchangeable positions for any position. The measurement system covers a wide range of analyses, including O and N. Furthermore, the detection and quantification of elements is performed from atomic number 4 (Beryllium). Finally, the electron microscope (FESEM) has a nitrogen injection charge compensation system. This system allows the observation of uncoated insulating samples using high vacuum electron detectors. The specimens analyzed by microscopy were covered with a thin layer of gold to give them conductive properties. In this way, it is possible to generate a beam of backscattered (e_1_) and secondary (e_2_) electrons.

**Figure 16:**
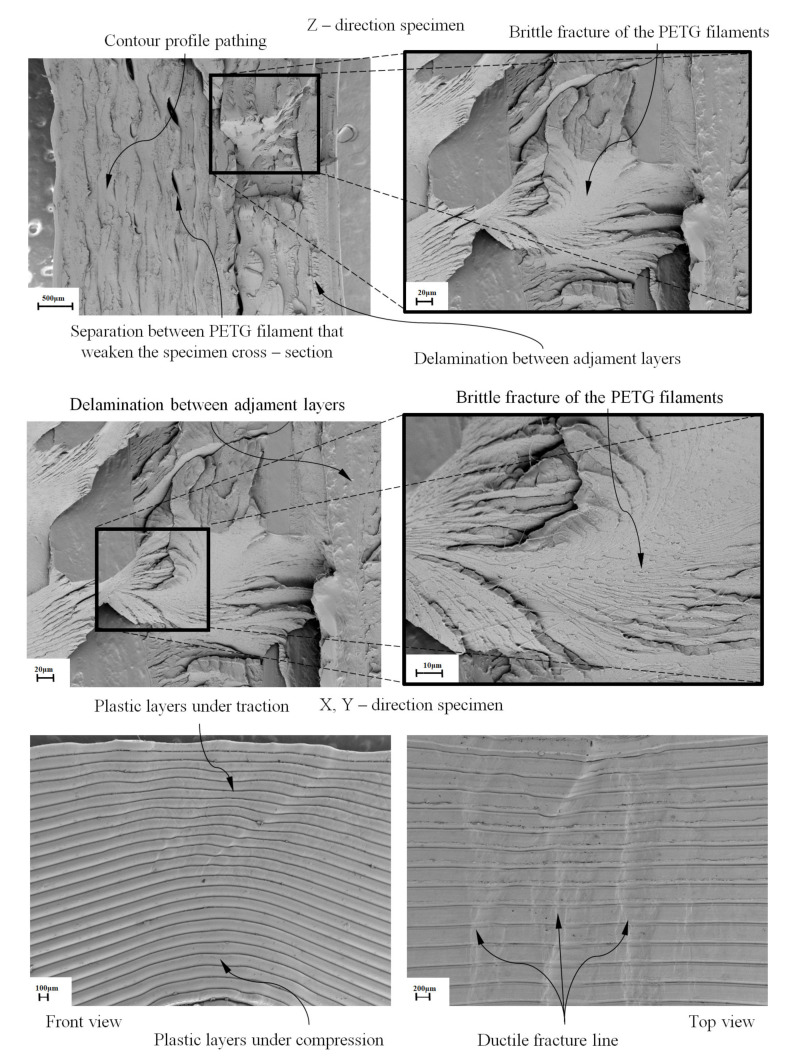
SEM images of the tested specimens.

To analyze the mechanics of the fracture produced in the test specimens, two specimens were selected (one for Z printing direction and the other for Y direction). Figure 16 (100–20, 20–10 and 100–200 μm) shows the results of two brittle fractures in a specimen manufactured in the Z direction. As can be seen, in both cases, the fracture mechanics are brittle, free, smooth and mainly produced by delamination between contiguous layers of welded filaments. The transverse and longitudinal fracture of the PETG filaments divides the original layers into several sub-layers that remain adhered to the adjacent layers after the fracture. The progress of the fracture progressively weakens the cross sections of the specimens, causing shear and bending stresses that affect the PETG filaments of the adjacent layers during the delamination process. As shown in Figure 16 (100–20, 20–10 and 100–200 μm), the longitudinal fracture of the PETG filament generates some very thin and long polymeric fibrils over the fractured surfaces. This could originate from a plastic fracture mechanism, causing the material to yield and form long PETG fibrils. Finally, the fibrils break and remain attached to the fractured surfaces.

Figure 16 (100–20, 20–10 and 100–200 μm) presents the permanent plastic deformation that occurs in the specimens printed in the X and Y directions after the experimental test. As can be seen in the profile view, during the plasticizing hardening process, the specimen undergoes a plastic deformation and a twist caused by a stress field due to a displacement which results in a specimen eccentricity. This fact causes uneven mechanical behavior between the layers of plastic material, since the upper layers (see Figure 16) are subjected to tensile stresses and the lower layers to compressive stresses. In this way, it is justified that the elastic behavior of the specimens printed in the X and Y direction is linear. When material reaches the yield stress point in a state of compression in the indicated directions of manufacture, PETG deforms in a plastic way, reaching a combined stress field between compression and flexion. The plan view for the X and Y printed specimens shows the perpendicular direction between the ductile rupture bands and the plastic strands under tension (see Figure 16). Specimens manufactured in X and Y are more flexible and less rigid, so they deform elastically and plastically until structural failure without fracturing the outer layers of material.

## 4. Conclusions

This paper presents the numerical and experimental analysis performed on the polymeric material PETG manufactured with FDM technology aiming at obtaining its mechanical characterization under uniaxial compression loads. Firstly, to evaluate the printing direction that presents the greatest mechanical strength, eighteen test specimens with prismatic geometry and rectangular cross section were manufactured according to the requirements of the ISO-604 standard. The mechanical behavior of the PETG plastic material for the test specimens manufactured in the X, Y and Z directions is completely linear until reaching the elastic limit at a compression value σ_c_, a point from which the plasticization process begins until the stress of fracture σ_f_. SEM micrographs showed different structural failures linked to the specimen manufacturing directions. For test specimens manufactured along the Z direction, the fracture was brittle, mainly caused by a delamination of adjacent layers and by a breakage of filaments. Conversely the specimens manufactured along the X and Y directions showed permanent plastic deformations due to material collapses presenting less strength compared to the specimens manufactured in Z.

A second experimental test analyzed the mechanical behavior of an innovative structural design manufactured in Z and X–Y directions under uniaxial compression loads according to the requirements of the Spanish CTE standard. The experimental curves of uniaxial compression forces versus nominal displacements showed different structural failures with regard to the manufacturing direction of the structural element. The structural element supported maximum uniaxial compression forces of 4942.0 N in Z and 2930.0 N in the X–Y manufacturing directions, these being admissible magnitudes considering the 780.4 N required by the Spanish CTE standard. The results ensure the structural safety of the element, validating the use of PETG material manufactured with FDM under the operational requirements presented.

A numerical validation of the structural part in both the Z and X–Y manufacturing directions according to the guidelines of the analytical model of the ISO-604 standard was performed. The compression Young’s modulus from the specimen tests were defined constant with values of 1329.5, 1117.9 and 1124.0 MPa, for the X, Y and Z manufacturing directions, respectively. Using the elastic and mechanical properties of the PETG material manufactured in the Z, X and Y directions and the requirements of the Spanish CTE standard, the numerical displacements for the structural element were 0.354, 0.412 and 0.414 mm in Z, X and Y, respectively. Thus, it can be said that the structural behavior of the structural element prototypes under the boundary stress conditions to which they will be subjected is far from collapse or fracture, validating the presented numerical model for PETG plastic material. The comparison between numerical and experimental results presented a percentage of relative error of 2.80% (Z-axis), 3.98% (X-axis) and 3.46% (Y-axis), which allows the characterization of the PETG plastic material manufactured with FDM as an isotropic material in the numerical simulation software without modifying the material modeling equations in the data software, thus validating the numerical model for the PETG plastic material presented in this paper.

The mechanical characterization of the polymer PETG and FDM technology presented by the authors was validated with a deviation of less than 4% between experimental and virtual results for all analyzed structural cases and according to the requirements of the ISO and CTE standards. This research is of great help to researchers working with polymers and FDM technology or companies that might need to numerically simulate new designs with PETG polymer and FDM technology since it is not currently possible to perform numerical simulations for geometries manufactured with PETG material using FDM technology requirements and subjected to pure compression loads due to the software being configured with mechanical behaviors of a standard isotropic and homogeneous PETG material. The research results indicate that the FDM process using PETG is encouraging for architectural applications, utilizing the advantages of the mechanical plastic properties and the freedom of building provided by the manufacturing process.

## Figures and Tables

**Table 1:** Main geometrical parameters of the plastic structural element.

Properties	Variable	Units	Value
Length	L	m	4.000
Thickness	T	m	0.045
Support width	W_s_	m	0.250
Support angle	α	^o^	42
Roof diameter	D	m	4.000
Panel thickness	T_p_	m	0.025
Panel length	L_p_	m	0.150

**Table 2:** Mechanical, physical properties and printing FDM features for PETG filaments.

Properties	Units	Value
Density	g/cm^3^	1.270
Notched izod impact	J/m^2^	105
Tensile strength	MPa	50
Flexural Modulus	MPa	2100
Flexural Strength	MPa	69
Rockwell Hardness	R scale	108
Vicat softening temperature	°C	85
Print temperature	°C	235 ± 10
Hot pad	°C	60–90

**Table 3:** Manufacturing parameters of the FDM process for the specimens and structural parts.

Manufacturing Parameters	Value	Units
Layer height	0.15	mm
Line width	0.3	mm
Contour lines	0.6	mm
Infill density	100	%
Overlap	30	%
Infill speed	48	mm/s
Wall speed	48	mm/s
Support pattern	Giroid	-
Support density	20	%
Support Z distance	0.2	mm
Support X–Y distance	0.75	mm
Nozzle size	0.4	mm
Infill pattern	Contour profile	-
Wall pattern	Contour profile	
Extrusion temperature	235	°C
Buildplate temperature	70	°C

**Table 4:** Test specimen dimensions for the characterization of PETG under compressive stress state.

Measurement Parameter	Length	Width	Thickness	Unit
Compression Young’s modulus	50 ± 2	10.0 ± 0.2	4 ± 0.2	mm
Compression yield stress	50 ± 2	10.0 ± 0.2	4 ± 0.2	mm

**Table 5:** Technical specifications of the compression test machine.

Model Number	Force Capacity (kN)	Vertical Test Space (mm)	Stiffness (N/m)
MTS–810	500	2108	7.5 × 10^8^

**Table 6:** Statistics of the mesh generated for the mechanical modeling.

Number of Elements	465,770
Nodes number	271,209
Quality of the element (Average)	0.889

**Table 7:** Elastic and mechanical properties of PETG for each Z-axis specimen.

Compression Properties	Units	S1	S2	S3	S4	S5	S6	Arithmetic Average	Typical Deviation
Young’s modulus, E_c_	MPa	1473.9	1207.1	1235.1	1244.6	1442.9	1373.4	1329.5	105.5
Yield stress, σ_y_	MPa	21.7	22.7	22.7	22.9	22.8	22.3	22.5	0.4
Fracture stress, σ_f_	MPa	18.6	19.2	18.4	17.4	18.2	16.9	18.1	0.8

**Table 8:** Elastic and mechanical properties of PETG for each X-axis specimen.

Compression Properties	Units	S1	S2	S3	S4	S5	S6	Arithmetic Average	Typical Deviation
Young’s modulus, E_c_	MPa	704.8	1091.1	899.2	1537.8	1140.0	1334.7	1117.9	271.7
Yield stress, σ_y_	MPa	23.7	23.4	20.9	20.5	10.0	18.9	19.6	4.6
Fracture stress, σ_f_	MPa	23.1	22.8	21.5	20.7	9.5	19.3	19.5	4.6

**Table 9:** Elastic and mechanical properties of PETG for each Y-axis specimen.

Compression Properties	Units	S1	S2	S3	S4	S5	S6	Arithmetic Average	Typical Deviation
Young’s modulus, E_c_	MPa	1504.3	937.7	1126.6	961.1	1254.4	959.94	1124.0	204.0
Yield stress, σ_y_	MPa	12.3	16.0	14.3	11.5	12.7	17.1	14.0	2.0
Fracture stress, σ_f_	MPa	12.9	16.3	14.7	11.6	12.9	17.5	14.3	2.1

**Table 10:** Mechanical properties for the printed element achieved in the uniaxial compression test on the Z-axis.

Properties of Compression	Units	Value
Uniaxial maximum force	N	4942.0
Nominal displacement at maximum uniaxial force	mm	3.623

**Table 11:** Mechanical properties for the printed element achieved in the uniaxial compression test on the X–Y-axis.

Properties of Compression	Units	Value
Uniaxial maximum force	N	2930.0
Nominal displacement at maximum uniaxial force	mm	1.928

**Table 12:** Comparison of the experimental and numerical results obtained on the structural element under the load scenario to which it is subjected.

3D Printing Direction	Experimental Displacement (mm)	Numerical Displacement (mm)	Relative Error (%)
Structural element-Z	0.365	0.354	2.789
Structural element-X	0.429	0.412	3.981
Structural element-Y	0.429	0.414	3.457
